# A quantitative method for the high throughput screening for the soil adhesion properties of plant and microbial polysaccharides and exudates

**DOI:** 10.1007/s11104-018-3670-1

**Published:** 2018-05-04

**Authors:** Jumana Akhtar, Andrew F. Galloway, Georgios Nikolopoulos, Katie J. Field, Paul Knox

**Affiliations:** Centre for Plant Sciences, Faculty of Biological Sciences, University of Leeds, Leeds, LS2 9JT UK

**Keywords:** Soil, Soil aggregate status, Polysaccharides, Soil-adhesion, Bioadhesives, Xyloglucan, Rhizosheaths

## Abstract

**Background and aims:**

Understanding the structures and functions of carbon-based molecules in soils is an important goal in the context of soils as an ecosystem function of immense importance. Polysaccharides are implicated in maintaining soil aggregate status but have not been extensively dissected in terms of their structures and soil adhesion properties. This is largely because of the technical difficulties in identifying polysaccharide structures and quantifying any functional properties.

**Methods:**

Here, we describe the use of a novel nitrocellulose-based adhesion assay to determine the relative capacities for soil adhesion of over twenty plant and microbial polysaccharides that are likely to be present in soil and to contribute to organic matter content and properties. Weights of soil adhered to spots of known amounts of specific polysaccharides were quantified by scanning of the nitrocellulose sheets.

**Results:**

The most effective polysaccharides identified from this survey included chitosan, β-1,3-glucan, gum tragacanth, xanthan and xyloglucan. We also demonstrate that the soil adhesion assay is suitable to assess the soil-binding properties of plant exudates.

**Conclusions:**

The soil adhesion assay will be useful for the functional dissection of the organic matter components of soils and also of the factors involved in soil attachment to plant roots and in rhizosheath formation.

## Introduction

Soils are an extremely important compartment of Earth’s ecosystems being crucial for plant growth and for maintaining the abundant and diverse biota of the terrestrial biosphere. Soils also play important roles in global carbon storage and cycling, feeding directly into regulation of the climate. Particle aggregation is key in influencing soil properties such as water and air flow and also in providing diverse niches for microbial and other biota. Declining soil health reflected in reduced aggregate size and soil compaction is an issue of global concern, not least with regard to agricultural soils and food security (Lal 1997; Kibblewhite et al. 2008; Shah et al. 2017).

The organic components of soil that are central to mineral particle adhesion and aggregation remain poorly characterised, being challenging to dissect in terms of precise identification of the molecules involved and their functional contributions (Cheshire et al. 1979; Lehmann and Kleber 2015). Organic matter derived from plant and microbial debris, mucilages and extracellular polymeric substances in general (such as cell walls and biofilms) are likely to influence the soil environment, potentially bioengineering local soil chemistry and properties to provide conditions conducive for specific needs (Oades 1984; Benizri et al. 2007; Lehmann and Kleber 2015). Polysaccharides have long been implicated in soil particle adhesion and aggregation but have not been dissected in detail due to the challenging nature of their diversity and structural complexities (Cheshire and Hayes 1990; Oades 1993). Major sources of soil polysaccharides will include plant debris and also plant secretions in exudates and mucilage (Walker et al. 2003; Dennis et al. 2010). Additionally microbial polysaccharides of bacterial and fungal origin as well as from animals such as earthworms (Wang et al. 2007) may contribute to mineral particle adhesion as well as acting as substrates and influencing microbial community dynamics. Polysaccharides have been proposed as being associated with large transiently stable aggregates and when present in exudates may work in balance with other factors to influence soil properties (Tisdall and Oades 1982; Naveed et al. 2017).

To date, scattered studies have added polysaccharides or root mucilages to soil samples to study factors such as aggregate stability and soil strengthening (Morel et al. 1991; Czarnes et al. 2000; Chang et al. 2015; Naveed et al. 2017; Galloway et al. 2018). A study using the wet sieving procedure has identified that the plant cell wall polysaccharide xyloglucan in particular can act as a potent soil aggregator (Galloway et al. 2018). While these studies have illuminated the potential role for plant and microbial polysaccharides in soil aggregation and adhesion the methods employed are relatively time-consuming.

An important feature of plant-soil interactions are plant influences on the rhizosphere and rhizosheath formation (Brown et al. 2017; Jin et al. 2017) although little is known about the role for any adhesive molecules at root surfaces. Methods are required to dissect the interface between plants and the soil environment and the roles of any secreted polysaccharides. Here, building on earlier work (Watt et al. 1993), we describe a simple and rapid assay in which, using commercially available polysaccharides, it is possible to screen for a polysaccharide’s ability, when adhered to nitrocellulose, to adhere soil particles. We also show that this soil adhesion assay is applicable to macromolecules secreted from plant roots. This methodology has the potential to significantly improve our understanding of the occurrence and activity of adhesive polysaccharides produced by soil organisms.

## Materials and methods

### Polysaccharides and collection and preparation of plant hydroponates/growth media

The commercial available polysaccharides assayed for soil adhesion properties are shown in Table 1. All polysaccharides were dissolved in dH_2_O apart from chitosan and alginic acid which were initially dissolved in 4 M KOH and then neutralised with 80% (*v*/v) acetic acid. Wheat (*Triticum aestivum* L. cv Cadenza), maize (*Zea mays* L. F1 cv. Earlibird), barley (*Hordeum vulgare* L. cvs Golden Promise), pea (*Pisum sativum* L. cv. Avola), and tomato (*Solanum lycopersicum* L. cv. Ailsa Craig) seeds were grown in vermiculite and perlite (50:50) for 7 d and then transferred to a hydroponic system which used 9 L containers and half-strength Hoagland’s growth media (H2395-10 L; Sigma-Aldrich, UK), topped up with distilled water when required, and with air (1 L/min) constantly pumped through. Twelve plants were grown in each container for 14 d with a 16 h photoperiod (light level of 1382 μmol m^−1^ s^−1^) at a constant temperature of 22 °C. The hydroponic media or hydroponates were passed through filter paper (Whatman Grade 2 V, 240 mm; GE Healthcare, Germany) to remove debris and then high molecular weight components were concentrated using a Centramate ultrafiltration system (FScentr005K10; PALL Life Sciences, US) with a 30 KDa cut-off point cassette that reduced each 9 L volume to ~200 ml using a flow rate of 500 ml/min. After concentration, the hydroponates were dialysed against dH_2_O (200× sample volume) for 3 d, with two changes per day, at room temperature using a 3.5 KDa cut-off membrane (Spectra/pro, Spectrumlabs, US). The dialysed concentrated hydroponates were transferred to 50 ml Falcon tubes (Falcon, US), and placed at −80 °C for 24 h and then lyophilised (Heto LyoPro 6000, US) to produce dry preparations of the high molecular weight components of the hydroponates. A liverwort, *Blasia pusilla* L., was grown in continuous shaking liquid culture as described (Galloway et al. 2018) and its growth media was collected and dialysed and lyophilised as described above for root exudates.

**Table 1:** Polysaccharides used in nitrocellulose-based soil adhesion assay

Polysaccharide	Class, species	Source
Arabinan (branched)	Plant, sugar beet	Megazyme (P-ARAB)
Arabinan (de-branched)	Plant, sugar beet	Megazyme (P-DBAR)
Gum Arabic	Plant, *Acacia* spp.	Sigma-Aldrich (G9752)
Gum karaya	Plant, *Sterculia* spp.	Sigma-Aldrich (G0503)
Gum tragacanth	Plant, *Astragalus* spp.	Sigma-Aldrich (G1128)
Mannan	Plant, ivory nut	Megazyme (P-MANIV)
Galactan	Plant, potato	Megazyme (P-GALPOT)
Pectin	Plant, apple (high methoxyl, >50%)	Solgar (033984001206)
Pectin	Plant, citrus (low methoxyl, 8%)	Sigma-Aldrich (P9135)
Polygalacturonic acid	Plant, citrus (no methoxyl)	Megazyme (P-PGACT)
Rhamnogalacturonan-I	Plant, potato	Megazyme (P-RHAM1)
Xylan	Plant, birch wood	Sigma-Aldrich (X0502)
Xyloglucan	Plant, tamarind	Megazyme (P-XYGLN)
Alginic acid	Brown agal/bacterial*, Macrocystis pyrifera*	Sigma-Aldrich (A7003)
Curdlan (carboxymethylated)	Bacterial	Megazyme (P-CMCUR)
Dextran	Bacterial, *Leuconostoc mesenteroides*	Sigma-Aldrich (D1537)
Levan	Bacterial*, Erwinia herbicola*	Sigma-Aldrich (L8647)
Xanthan	Bacterial*, Xanthomonas campestris*	Sigma-Aldrich (43708)
Chitosan	Fungal, White mushroom	Sigma-Aldrich (419419)
Fucoidan	Fungal*,* algal proxy from *Fucus vesiculosus*	Sigma-Aldrich (F5631)
Pachyman (carboxymethylated)	Fungal, *Poria cocus*	Biosupplies Australia (300-2)

### Nitrocellulose-based polysaccharide-soil adhesion assay

**Fig. 1:**
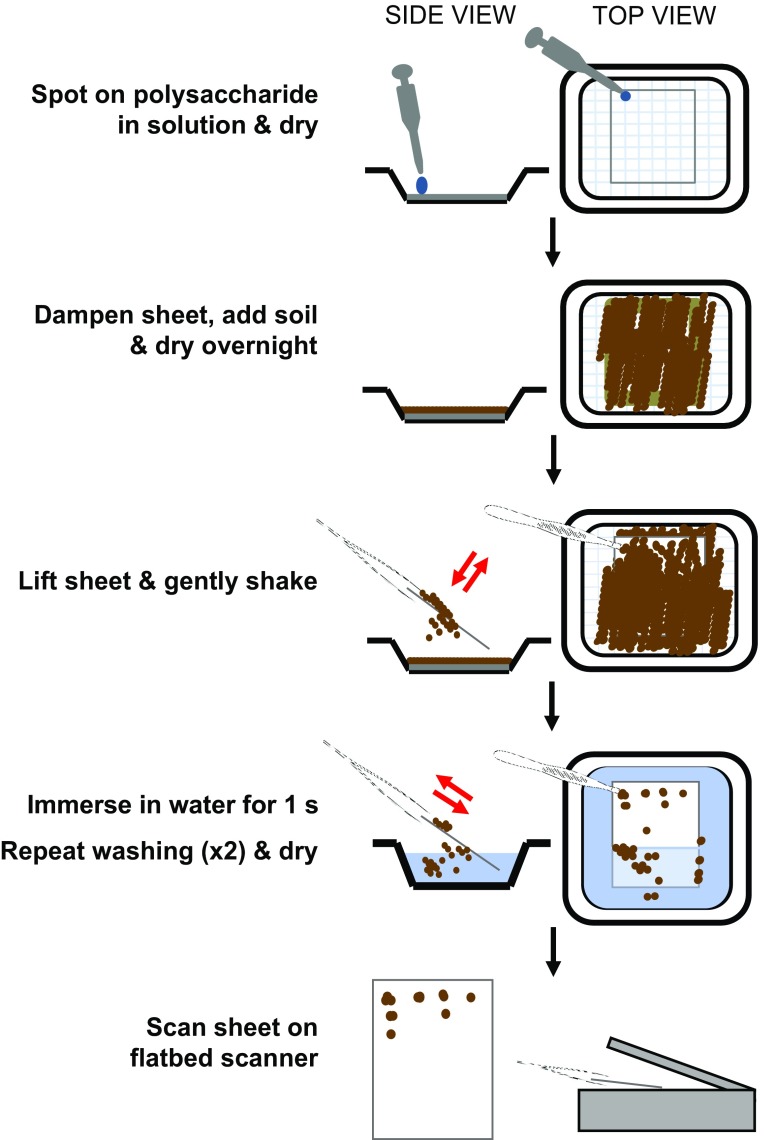
Schematic outline of major steps in the soil adhesion assay protocol showing application of polysaccharides to nitrocellulose sheets which are then dried. Before use the sheets are re-dampened and then overlain with soil and left to dry again. Sheets are then carefully removed, gently shaken and washed by immersion twice before a final drying and scanning. A calibration curve is used to convert Image J mean grey values to weights of bound soil

Our novel nitrocellulose-based polysaccharide-soil adhesion assay is depicted stepwise in Fig. 1. Pieces of dry nitrocellulose membrane sheets (Amersham Protran 0.45 μm, GE Healthcare, Germany), marked with 1 cm^2^ squares, were placed in 10 cm × 10 cm weighing boats and placed on a light box. Polysaccharide solutions in 5 μl aliquots (usually starting at 50 μg/5 μl and down to 0.4 μg/5 μl and a final spot of 5 μl distilled water) were applied to the centre of the marked squares so that several polysaccharides were present on the same sheet. The weighing boats were covered loosely with foil and the sheets left to air-dry for 2 h at room temperature. The nitrocellulose sheets could be stored in this form before use for at least a week. Immediately before use, nitrocellulose sheets were immersed in distilled water for 5 s, excess water removed by gentle shaking and then placed in a fresh weighing boat and immediately the whole sheets were evenly covered to a 2 mm depth with sterile, air-dried, sieved ≤500 μm top soil. The weighing boats were covered in foil and left to air dry overnight which was found to be an effective period to maximise soil adhesion. When dry, the nitrocellulose sheets were picked up with tweezers and gently shaken to remove soil. Individual sheets were then immersed in 200 ml of distilled water for 1 s (twice) and then the moist nitrocellulose sheets were placed in a fresh weighing boat and allowed to air dry for at least 3 h before analysis.

Nitrocellulose sheets with adhered soil were scanned with an Epson Perfection V750 Pro scanner using Epson Scan professional mode (resolution set at 1200 dpi, tone curve input 181/output 199). Image J was then used to quantify the soil adhered to each spot as follows. A circle was drawn around each spot and the mean grey value was derived and the difference from the mean grey value from the control (no polysaccharide) spot was taken as a measure of the adhered soil. As the individual polysaccharides spread to different extents on the nitrocellulose sheet a circle was drawn for the appropriate area for each polysaccharide. Using effective soil binders gum tragacanth and xyloglucan the relationship of mean grey value to the weight of adhered soil was determined. Known amounts of polysaccharide (in range 0.4 to 50 μg per spot) were spotted on to individual 20 mm × 20 mm squares of nitrocellulose sheets and the soil adhesion assay carried out with the nitrocellulose pieces with single spots of polysaccharide being weighed before and after soil binding. In this analysis the regression coefficient was 0.9716 and a calibration curve equation (Weight soil bound (mg) = 0.0138*mean-grey-value – 0.0248) was used to convert mean grey values to weight of soil bound per spot. All assays were done at least in triplicate. The statistical significance of differences between values was determined using the Mann-Whitney U test. For data sets with three or more treatments a Kruskal-Wallis test was followed by post-hoc Mann-Whitney U tests. Differences were considered significant when the *p*-values were below 0.05.

## Results

### Screening of polysaccharides for soil-adhesive properties

A panel of 21 commercially available polysaccharides was assembled (Table 1). This included a range of plant-derived pectic polysaccharides and gums including gum Arabic that contains arabinogalactan-protein-like structures that are found in root mucilage (Bacic et al. 1986; Moody et al. 1988; Verbeken et al. 2003; Koroney et al. 2016). Additionally, we included non-pectic, non-cellulosic cell wall polysaccharides including heteroxylan, heteromannan and xyloglucan that are major components of plant debris and with heteroxylans been abundant in grass/cereal biomass. Available bacterial polysaccharides tested included xanthan, curdlan and levan and we also included alginic acid (from brown algae) that can be produced by *Pseudomonas* species (Chang et al. 2007). Fungal polysaccharides included chitosan, the 1,3-glucan pachyman and the brown alga fucoidan as a proxy for the sulphated fucan polysaccharides recently identified in cell walls of mucoromycotina fungi (Mélida et al. 2015).

**Fig. 2:**
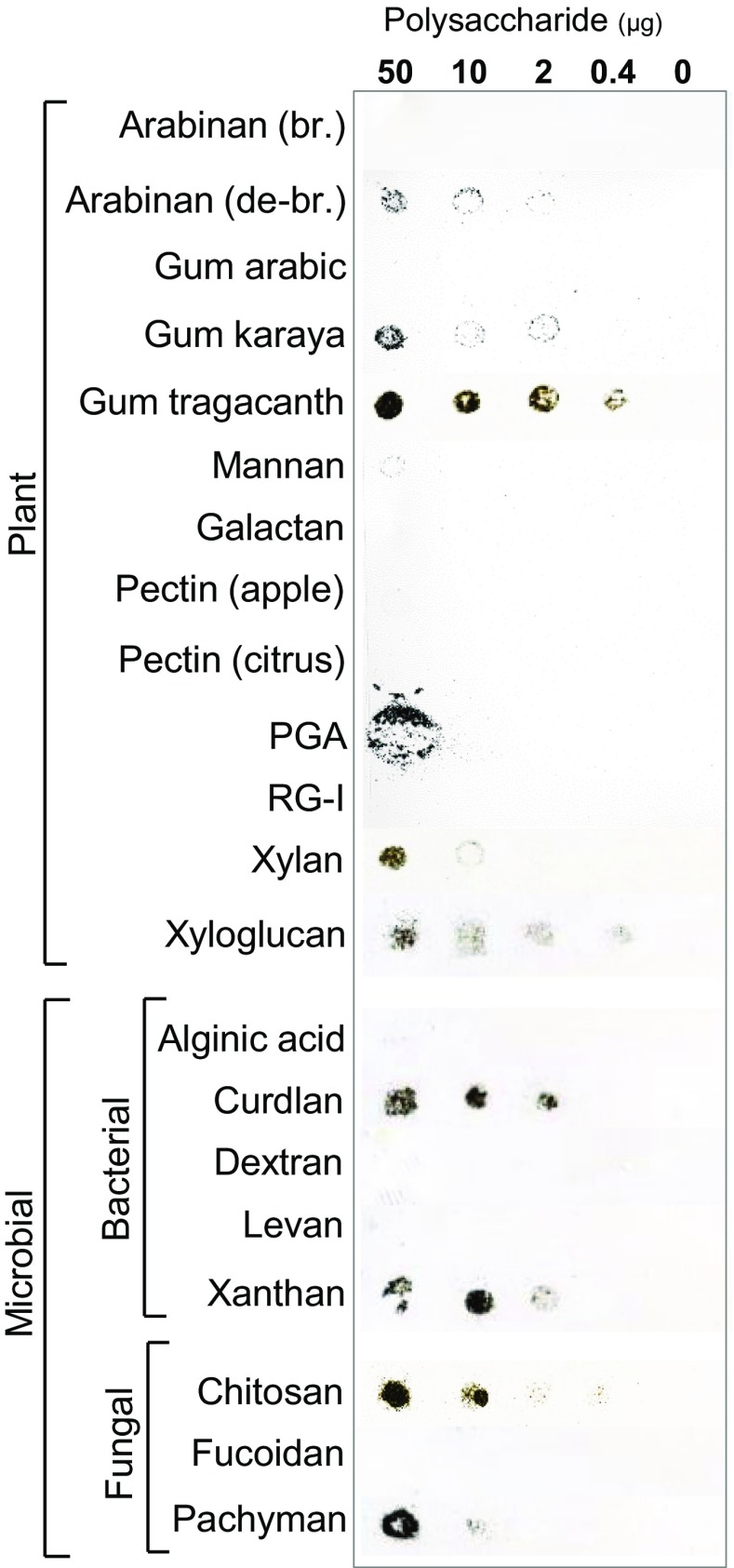
**High throughput analysis of the soil adhesive properties of commercial polysaccharides.** Representative nitrocellulose sheets showing twenty-one commercial polysaccharides (plant, bacterial and fungal) spotted with 5 μL dots and after sieved soil (<500 μm) was laid on the moistened sheets and left overnight. After incubation, nitrocellulose sheets were washed in distilled water, air-dried prior to scanning to determine the weights of bound soil. PGA = polygalacturonic acid, RG-I = rhamnogalacturonan-I from potato br. = branched and de-br. = debranched

To determine the adhesive properties of these polysaccharides they were first immobilised on nitrocellulose membrane sheets - a proven substrate for the immobilization of biological polymers and widely used for the detection of polysaccharides and proteins by molecular probes such as antibodies. Dissolved polysaccharides were applied to dry nitrocellulose membrane and allowed to dry completely. The sheets were then moistened and overlaid with dry soil and left to dry. Sheets were then removed, dipped twice in water and then dried and scanned and scanned values converted to weights of soil bound per spot. A schematic outline of the procedure is shown in Fig. 1 and examples of soil-treated nitrocellulose sheets are shown in Fig. 2. It can be seen that soil bound effectively to the spots of applied polysaccharides and also that there was a clear variation in the capacities of the different polysaccharides tested to adhere soil particles. Most polysaccharides were contained on the nitrocellulose in small spots and soil adhered to gum tragacanth, curdlan, xyloglucan and chitosan when applied at 0.4 μg/spot. Polygalacturonic acid (PGA) was notable for its spreading in a larger spot (Fig. 2, see Willats and Knox (1999) for a discussion of its behaviour when applied to nitrocellulose) and was effective in soil adhesion although only at the highest concentration applied (50 μg/spot).

**Fig. 3:**
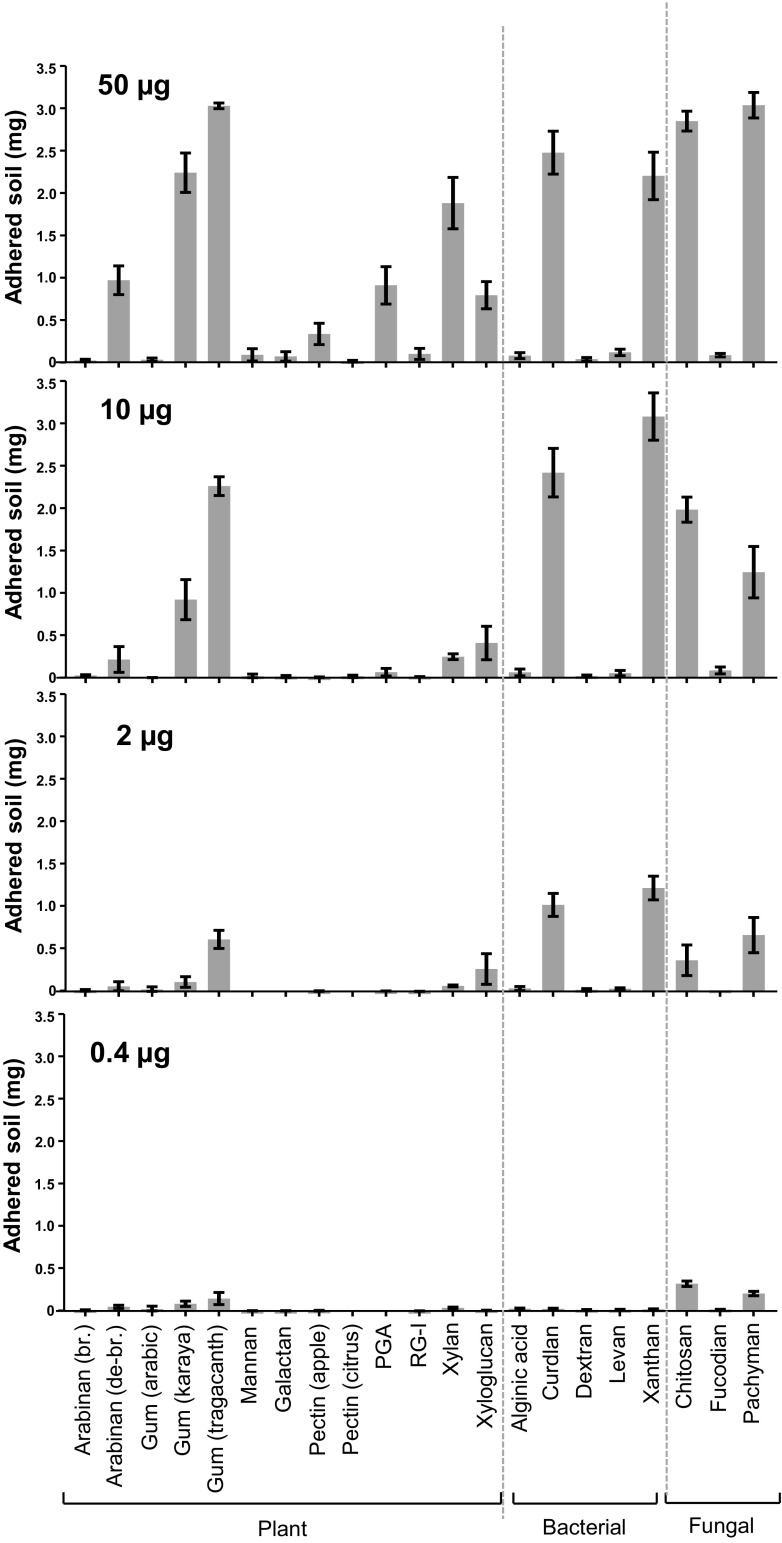
**Quantification of soil adhered to nitrocellulose-immobilised polysaccharides.** The amount of soil adhered to spots of 21 commercial polysaccharides immobilized on nitrocellulose sheets (see Fig. 2 for an example) using 50 μg to 0.4 μg polysaccharide/spot as shown. Bound soil was quantified by use of a calibration curve of the Image J mean grey value against weight of soil in mg/spot. Each data point is a mean of three biological replicates and error bars = SD. PGA = polygalacturonic acid, RG-I = rhamnogalacturonan-I from potato br. = branched and de-br. = debranched

The nitrocellulose-based soil adhesion assay was found to be repeatable and consistent when the adhered soil in replicate experiments was quantified as shown in Fig. 3. Most polysaccharides were proportionally effective across the concentration range assayed, although three plant cell wall polysaccharides likely to be abundant in soils PGA, heteroxylan and xyloglucan - varied in this regard with the effectiveness of PGA and heteroxylan at higher concentrations not being apparent at lower concentrations relative to xyloglucan. This may indicate a specific property of xyloglucan in terms of bioadhesion. It is also of note that the debranched form of arabinan was more effective that branched arabinan (Figs. 2 and 3).

For the microbial polysaccharides it was notable that both the fungal and bacterial β-1,3-glucans (pachyman and curdlan) were effective adhesives and also that chitosan of fungal origin and xanthan of bacterial origin were effective at the lower concentrations.

### Soil-adhesive properties of materials released from plants

**Fig. 4:**
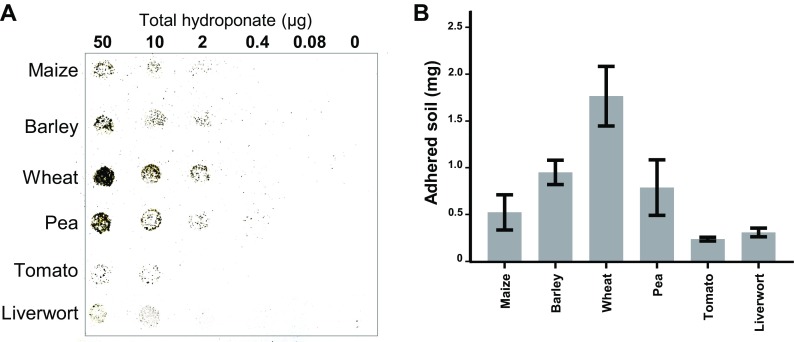
**Analysis of the soil adhesive properties of high molecular weight plant exudates.** A range of plants were grown hydroponically and the isolated high molecular weight (HMW) fractions of the hydroponates were spotted (5 μL dots) onto nitrocellulose sheets and air-dried. Sieved soil (<500 μm) was laid on moistened nitrocellulose and left overnight. After incubation, nitrocellulose sheets were washed in distilled water, air-dried and weights of adhered soil determined. **a**. Shows a representative single sheet of nitrocellulose with soil adhering to exudates at a range of concentrations. **b**. Quantification of soil adhered when HMW exudates applied at 50 μg/spot. *n* = 3, error bars = SD

To extend the method from its use in the assay of the adhesive properties of isolated commercially-obtained polysaccharides, we also explored the assay to determine the adhesive properties of plant root and liverwort rhizoid exudates obtained from hydroponic systems as shown in Fig. 4. The high molecular weight (HMW) components of the exudates were isolated by dialysis and lyophilisation and applied in the same range of concentrations as used for the individual polysaccharides. These preparations of HMW hydroponate components have previously been screened for the presence of cell wall related polysaccharides using monoclonal antibodies (Galloway et al. 2018). There was a clear soil adhesive capacity for the HMW exudates when applied to nitrocellulose down to 2 μg/spot for wheat, barley and pea roots and for all exudates at the 10 μg/spot and above.

### Concentration dependency of soil binding properties

**Fig. 5:**
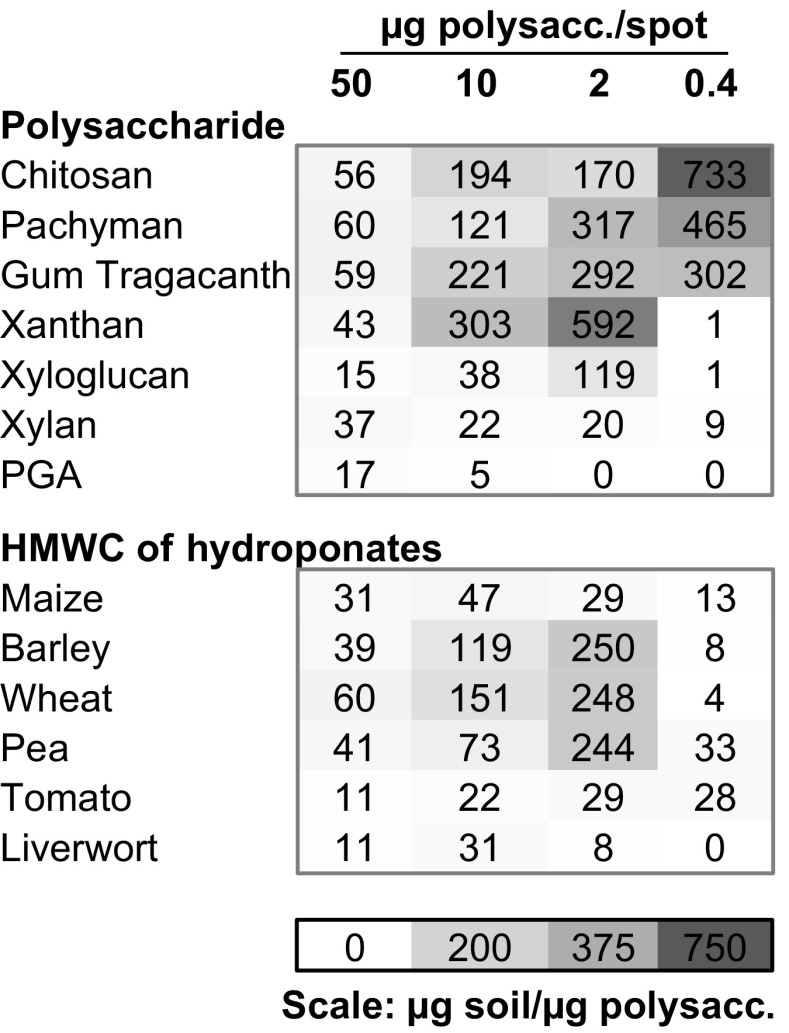
**Heat map of soil binding capacities of polysaccharides and hydroponates expressed on a weight/weight basis.** Data shown for four levels of application to 5 μl spots on nitrocellulose sheets and expressed as μg soil bound per μg polysaccharide

An analysis of bound soil expressed as μg soil/μg polymer across the four levels of application to spots on nitrocellulose sheets for selected polysaccharides and the hydroponate preparations is shown in Fig. 5. This clearly demonstrates the strong soil-binding properties of specific polysaccharides. Moreover, the effectiveness of the assay is demonstrated by some of the lower levels of polysaccharide application and the associated quantitation of the adhered soil. For example, chitosan at 0.4 μg/spot resulted in the adhesion of >700 μg soil per μg chitosan and pachyman >400 μg/μg at the same level of application. It is of interest that for chitosan and pachyman the most effective level of application to the nitrocellulose was at 0.4 μg/spot whereas gum tragacanth had a similar effectiveness at 2 μg/spot and 0.4 μg/spot levels. In the case of xanthan, it was most effective of all tested polysaccharides at the 2 μg/spot application level and bound ~600 μg soil/μg and yet virtually none at the 0.4 μg/spot level. These polysaccharides all bound soil at 60 μg/μg at the highest level of application (50 μg/spot) suggesting a maximal amount of soil being able to be bound to a 5 μl spot. The differences between polysaccharides observed at lower levels of application would appear to reflect varied aspects of molecular crowding and the varied spreading and distribution of polymers within spots as they become immobilised on the nitrocellulose substrate.

The same analysis the HMWC of the hydroponates indicated that barley, wheat and pea were most effective at the 2 μg/spot level (binding ~250 μg soil/μg) whereas the others were more evenly effective across the application levels. The most effective soil-binding exudate was that obtained from wheat and the application of 50 μg of total HMW hydroponate to a spot on nitrocellulose resulted in the binding of 60 μg soil per μg – the same capacity as the most effective individual polysaccharides.

## Discussion

Understanding the function of plant-secreted polysaccharides as a component of the organic matter of soils is an important factor in understanding soil properties and health. Here, using a novel method of immobilised polymers on nitrocellulose sheets we show that not all polysaccharides are equal in terms of their abilities to adhere soil particles. Our newly developed, high throughput soil adhesion assay extends and confirms recent assessments using routinely-used wet sieving methods that xyloglucan is more effective than pectic HG and gum Arabic in promoting soil particle adhesion (Galloway et al. 2018) and identifies effective soil-binding polysaccharides of microbial origin. The soil adhesion assay is also of interest in that as the polymers are immobilised, it can also be considered as a potential mimic of polysaccharides likely to found at plant root and rhizoid surfaces and also in biofilms produced by microorganisms. As such, the assay may find use as a screen for factors that are involved in the formation and stabilization of rhizosheaths that are important in some groups of plants for the optimization of water uptake and survival in drought conditions (Watt et al. 1994; Carminati and Vetterlein 2013; Haling et al. 2014; Brown et al. 2017). It will be important to further dissect the bio-adhesive properties of macromolecules in plant exudates as shown here.

This initial survey of a range of polysaccharides does not readily allow soil adhesion effectiveness to be directly related to specific carbohydrate structural features. The most effective polysaccharides in this assay were chitosan, 1,3-glucan and also gum tragacanth from *Astragalus* species which is a complex heterogeneous highly branched anionic polysaccharide of high molecular weight (Verbeken et al. 2003; Balaghi et al. 2011). Although pectic PGA was effective at the highest concentration, other acidic pectins including rhamnogalacturonan-I and pectins with higher degrees of methylesterification and alginic acid were not. A notable feature of the results is that debranched arabinan was more effective than branched arabinan and that two 1,3-glucans were effective as was xanthan which has a 1,4-glucan backbone with side chains of mannosyl and glucuronosyl residues that can also carry acetyl and pyruvate groups. The effectiveness of polysaccharides as bio-adhesives is therefore likely to reside in properties such as size, conformation, hydration properties and viscosities. Polysaccharide preparations can be highly heterogeneous and factors such as size can be challenging to determine. Here we have developed the soil adhesion assay using a diverse range of polysaccharides that has revealed clear differences in their soil-binding properties. Future work assessing the soil-binding properties of sets of related polysaccharides with, for example, defined degrees of polymerization or, in the case of pectin, degrees of methylesterification will be of interest.

The soil adhesion assay described here will be useful to dissect the polysaccharides found at plant surfaces that interface with soils and in also soils in general. Future work should aim to both define the bio-adhesives in plant exudates/secretions and also be widened to include assessments of polysaccharides from other sets of soil organisms. An interesting aspect for future work will be determining any synergies arising from mixtures of polysaccharides. It is of interest in this context that different polysaccharides have differential capacities to bind soil dependent on concentration. It is possible that multi-component sets of soil-binding polysaccharides are released by plants or other organisms that can regulate the overall binding to the highly heterogeneous substrates that are soils. Functional dissection of soil polysaccharides in general may lead to the development of soil glycotyping as a useful methodology to understand soil carbon contents and dynamics and to influence soil management practices – potentially through the addition of plant or other residues to improve soil structure and function.
